# Rs2015 Polymorphism in miRNA Target Site of Sirtuin2 Gene Is Associated with the Risk of Parkinson's Disease in Chinese Han Population

**DOI:** 10.1155/2019/1498034

**Published:** 2019-05-12

**Authors:** Xiongjin Chen, Hui Mai, Xiaoting Chen, Yujie Cai, Qiufei Cheng, Xiaoyi Chen, Xiaohui Li, Weihao Fan, Pei Tang, Mingqian Ou, Jingqi Yang, Yusen Chen, Lili Cui

**Affiliations:** ^1^Guangdong Key Laboratory of Age-Related Cardiac and Cerebral Diseases, Affiliated Hospital of Guangdong Medical University, Zhanjiang, Guangdong, China; ^2^Institutes of Neurology, Affiliated Hospital of Guangdong Medical University, Zhanjiang, Guangdong, China

## Abstract

Accumulating evidence reveals that the sirtuin family is involved in the pathology of Parkinson's disease (PD). However, the association between the polymorphisms of the sirtuin gene and the risk of PD remains elusive. Here, we investigated the possible association of nine SIRT1 and SIRT2 SNPs with the risk of PD through a clinical case-control study from the Chinese Han population. Our results showed that rs12778366 in the promoter region of SIRT1 and rs2015 in the 3′untranslated region (3′UTR) of the SIRT2 were significantly associated with the risk of PD. Five SNPs related to SIRT1, rs3740051, rs7895833, rs7069102, rs2273773, and rs4746720 and two SNPs related to SIRT2, rs10410544, and rs45592833 did not show an association with PD risk in this study. Moreover, we found that mRNA level of SIRT2 was upregulated, and mRNA level of SIRT1 was downregulated in the peripheral blood of PD patients compared with healthy controls, and we also observed that SNPs rs12778366 and rs2015 influenced the SIRT1 and SIRT2 expression levels, respectively. Further functional assays suggest that rs2015 may affect the expression of SIRT2 by affecting the binding of miR-8061 to the 3′UTR of SIRT2, ultimately contributing to the risk of PD.

## 1. Introduction

Parkinson's disease (PD) is the second most common neurodegenerative disorder with existing treatments being only symptomatic and cannot prevent disease progression [1]. PD is clinically characterized by rigidity, resting tremors, and bradykinesia [2], pathologically characterized by the presence of Lewy bodies (LB); the main component is accumulated misfolded *α*-Synuclein (*α*-Syn) [3, 4] and progressive loss of dopaminergic neurons in the substantia nigra pars compacta (SNc) [5, 6]. The genetic study about rare inherited forms of PD has given important clues to the molecular pathogenesis of the disease, but the pathogenesis of PD is still unclear [7]. For more than 90% of PDs are sporadic and late-onset, it is likely that for most cases, there is a complex interplay between environmental and genetic factors influences in the onset of PD [8]. There are now a large number of published* genome-wide association (GWA)* studies about PD suggesting new risk factors for PD in different population [9].

Sirtuins are NAD+-dependent deacylases which play a vital role in various physiological functions and diseases progression [10], especially governing the effects of the brain on ageing [11]. Manipulating activities of SIRT1 and SIRT2 show the opposing effects in neurodegenerative disease [12]. Activation of SIRT1 has protective effect on PD which is similar to the results with the inactivation of SIRT2 [13]. SIRT1 expression was found to be markedly decreased in multiple PD model, induced either by environmental factor or by genetic factor [14]. The activity of SIRT1 was observed to be downregulated in patients with PD and other neurodegenerative disease patients [15]. Overproduction of SIRT1 has been showed to protect SH-SY5Y cells from toxin induced cell death and mitigate the *α*-Syn aggregation in cell and animal models of PD [16]. The SIRT1 activator resveratrol was demonstrated to have a protective effect against rotenone-induced apoptosis in SH-SY5Y cells [17] and *α*-Syn-induced toxicity in SK-N-BE cells [18].The inhibitor or genetic knockout of SIRT2 can restore behavioral deficits in 1-methyl-4-phenyl-1, 2, 3, 6-tetrahydropyridine (MPTP) treated aging mice [19, 20]. Furthermore, SIRT2 deletion mice could reduce MPTP-induced nigrostriatal damage; meanwhile silencing or overexpressing SIRT2 was also positively associated with apoptosis in MPP+-treated SH-SY5Y cells [21]. In a human cell line model of *α*-Syn inclusion formation, pharmacological inhibition of SIRT2 attenuated the *α*-Syn toxicity [22]. Recently, the mechanism has been uncovered; genetic manipulation of SIRT2 levels induces *α*-Syn acetylating, thereby reducing *α*-Syn aggregation and increasing autophagy [23].

The above evidence supports the involvement of SIRT1 /SIRT2 gene in PD progress, but there is still lack of genetic data evaluating SIRT1 and SIRT2 as PD risk factors in the Chinese Han population. In the study, we endeavored to shed light on the possible association between the single nucleotide polymorphisms (SNPs) in the SIRT1/SIRT2 gene and susceptibility to PD in the Chinese Han population for the first time. Furthermore, the mechanism by which the SNP affects PD susceptibility was preliminarily analyzed.

## 2. Materials and Methods

### 2.1. Subjects

This study consisted of 222 PD patients (mean age±SD, 68.17 ± 10.06 years; male/female = 134/88) and 161 control subjects (mean age±SD, 66.34 ± 7.50 years; male/female = 89/72). All PD patients were enrolled from the Affiliated Hospital of Guangdong Medical University (Zhanjiang, China) between August 2006 and August 2017. A complete anamnesis in accordance with the United Kingdom brain-bank criteria from two or more experienced neurologists was collected to ensure that all the patients in the study suffered from PD presenting parkinsonian motor features, namely, bradykinesia plus rigidity and resting tremors. Patients who had a history of or suffered from atypical parkinsonism, including progressive supranuclear palsy, multiple system atrophy, vascular or drug-induced parkinsonism, and severe dementia, were excluded from our study. Healthy controls were also examined by neurologists, who excluded related neurologic disorders, cardiovascular diseases, and the presence of any movement disorder, particularly PD. Both cases and controls were of Chinese Han descent. All participants gave informed consent. This study was conducted following the Declaration of Helsinki and under the approval of the ethics committee of the Affiliated Hospital of Guangdong Medical College.

### 2.2. Genotyping

A fasting venous blood sample of 2 ml was extracted from each subject at the elbow on the morning after diagnosis and was treated with ethylenediaminetetraacetic acid dipotassium salt (EDTA-K2) to prevent coagulation. Within 1-4 h, 0.5 ml of the blood sample was added to two 1.5 ml EP tubes. Genomic DNA was extracted from 2 ml peripheral blood samples collected from each participant using a Blood Genomic DNA Extraction Kit (Tiangen, China). The SIRT1/SIRT2 genetic variants were genotyped using the SNaPshot Multiplex Kit (Genesky Biotechnologies, China). The reaction system consisted of 5 *μ*l SNaPshot Multiplex Kit (ABI, Shanghai), 2 *μ*l template DNA, 2 *μ*l ddH_2_O, and 1 *μ*l primer mix. The SNaPshot response program included 95°C for 2 minutes and 94°C for 20 s (11 cycles, 65°C for 40 s and 72°C for 90 s). Then, the extension products were purified via incubation with 1 U of shrimp alkaline phosphatase (Takara, Japan) for 15 minutes at 37°C and subsequent incubation at 80°C for 15 minutes to inactivate the enzyme. Additionally, the purified products (0.5 *μ*l) were mixed with 9 *μ*l of Hi-Di formamide and 0.5 *μ*l of the Lizl20 Size Standard (Applied Biosystems, USA) and separated in the ABI Prism 3730XL Genetic Analyzer (Applied Biosystems, USA). The final data were analyzed with GeneMapper 4.1 (Applied Biosystems, USA).

### 2.3. Cell Culture

The HEK-293T cell lines were obtained from the Shanghai Cell Institute Country Cell Bank (Shanghai, China). The cell lines were grown in Dulbecco's Modified Eagle's Medium (DMEM) supplemented with 10% fetal bovine serum (FBS) (Gibco, Australia), 100 U/ml penicillin G, and 100 ug/ml streptomycin (Hyclone, Shanghai). The cells were maintained at 37°C in a humidified 5% CO2 incubator during growth and treatment. SH-SY5Y cells purchased from Genechem Inc. (Shanghai, China) were cultured in complete DMEM supplemented with 10% FBS and 1% penicillin-streptomycin antibiotics at 37°C in 5% CO2. Confluent cell cultures were passaged with 0.25% trypsin containing ethylenediaminetetraacetic acid (EDTA).

### 2.4. Plasmid Constructs

To build luciferase reporter constructs carrying the 3′UTR of SIRT2, 614 bp in the 3′UTR of SIRT2 was amplified and cloned into the psiCHECK-2 luciferase vector using the restriction enzymes XhoI/NotI (IGEbio, Guangzhou). Luciferase reporter constructs including the wild/mutant rs2015 genotype were created by the QuikChange® Site-Directed Mutagenesis Kit (Stratagene, Shanghai). After transformation into* Escherichia coli* DH5a cells, all of the plasmids were isolated and purified using a Plasmid Midi Kit (Promega, USA). The constructs were confirmed by sequencing.

### 2.5. Luciferase Assay

HEK-293T cells were transiently transfected for 48 h with the firefly luciferase psiCHECK-2 haplotype reporter and Renilla luciferase psiCHECK-2 vectors using Lipofectamine 2000 (Invitrogen, USA) according to the manufacturers' instructions. Three parallel samples were used in all transfections, and all experiments were performed in triplicate. The assays were performed according to the protocol of the dual luciferase assay kit (Beyotime, Shanghai). The luminescence was measured using a Mithras LB940 Multilabel Reader (Berthold Technologies, Bad Wildbad, Germany). The activity of Renilla luciferase was normalized to that of firefly luciferase.

### 2.6. Western Blotting

Western blotting was performed according to standard western blotting procedures. The harvested SH-SY5Y Cells were lysed in NP-40 buffer containing protease inhibitor cocktail (Sigma, USA) and 1 mM phenylmethylsulfonyl fluoride (Sigma, USA). Lysates were centrifuged at 12,000 g for 15 minutes at 4°C. Supernatants were collected, and protein concentrations were determined by the BCA Protein Assay Kit (Thermo, USA). Proteins were then separated via 10% SDS-PAGE and transferred to a polyvinylidene fluoride (PVDF) membrane (Millipore, USA). After blocking in 5% nonfat milk, the membranes were incubated with the following primary antibodies: a-tubulin (Abcam; 1:300) and anti-SIRT2 (Abcam; 1:1000). The proteins were visualized with enhanced chemiluminescence reagents (Pierce, Shanghai) in the machine (Azure Biosystems, USA).

### 2.7. RNA Extraction and Quantitative Real-Time Reverse Transcription PCR (qRT-PCR)

Total RNA was extracted from peripheral blood leukocytes or cultured cells using the TRIzol reagent (Invitrogen, USA), while the cDNA for SIRT2 detection was synthesized with the PrimeScript TM RT reagent kit (Takara, Japan) according to the manufacturers' instructions. Furthermore, the cDNA used to evaluate miR-376a-5p/miR-4760-5p/miR-8061 was synthesized using the miRcute miRNA cDNA First-Strand Synthesis Kit (Tiangen, China) according to the manufacturer's instructions. The expression of SIRT2 with GAPDH served as an internal reference, and the expression of miR-376a-5p/miR-4760-5p/miR-8061 was examined using SYBR® Premix Ex Taq TM II (Takara, Japan), with U6 serving as an internal reference. The PCR primers were as follows: hsa-miR-376a-5p F, 5′ CCCTCGATGTAGATTCTCCTTC 3′; hsa-miR-376a-5p R, 5′ TATGCTTGTTCTCGTC -TCTGTGTC 3′; hsa-miR-4760-5P F, 5′ CCCACGATTTTAGATTGAACAT 3′; hsa-miR-4760-5P R, 5′ TATGCT -TGTTCTCGTCTCTGTGTC 3′; hsa-miR-8061 F, 5′ TCGC -TCCATCTTAGATTAGAGG 3′; hsa-miR-8061 R, 5′ TATGCTTGTTCTCGTCTCTGTGT -C 3′; SIRT2 F, 5′ TAGATACCCTGGAGCGAATAG 3′; and SIRT2 R, 5′ AC -ACTTGGGCGTCCTC 3′. All experiments were carried out in triplicate. All implied primers were purchased from IBSBIO (Shanghai, China).

### 2.8. Statistical Analysis

All SNPs were evaluated for Hardy-Weinberg equilibrium (HWE) using the chi-square test. The chi-square test was used to compare allele and genotype distributions between cases and controls. The odds ratio (OR) and corresponding 95% confidence interval (CI) were calculated. The means between two groups were compared with unpaired, two-tailed Student's t test. Data were analyzed with GraphPad Prism 6 software. P values <0.05 were considered statistically significant.

## 3. Results

In this study, 222 PD patients and 161 control subjects were enrolled according to strict inclusion criteria (Table 1). No significant differences in age or gender distribution were observed between the cases and the controls (P=0.0581 and 0.3196, respectively). All of the genotype distributions followed Hardy-Weinberg equilibrium (HWE) in the cases and controls (all P>0.05). The genotype and allele frequencies of these nine SNPs and their associations with the risk of PD are listed in Table 2. A significant association was observed in the genotype and allele frequencies of the rs12778366 SNP between the PD cases and controls (genotype P=0.0459 and allele P=0.0111). A significant difference in the genotype frequencies of the rs2015 SNP between PD patients and controls was also observed (P=0.0431). Moreover, rs12778366 T>C and rs2015 G>T both showed a significant difference in cases compared with the controls in the dominant model, suggesting that the two SNPs may be a risk factor for PD in Chinese Han population (P=0.0221 and P=0.0436, respectively). With regard to the genotype and allele frequencies of the other seven polymorphisms (rs3740051 A>G, rs2273773 T>C, rs7069102 C>G, rs4746720 T>C, rs7895833 G>A, rs10410544 C>T, and rs45592833 G>T), no significant associations were found between the case and control groups (Table 2).

**Figure 1:**
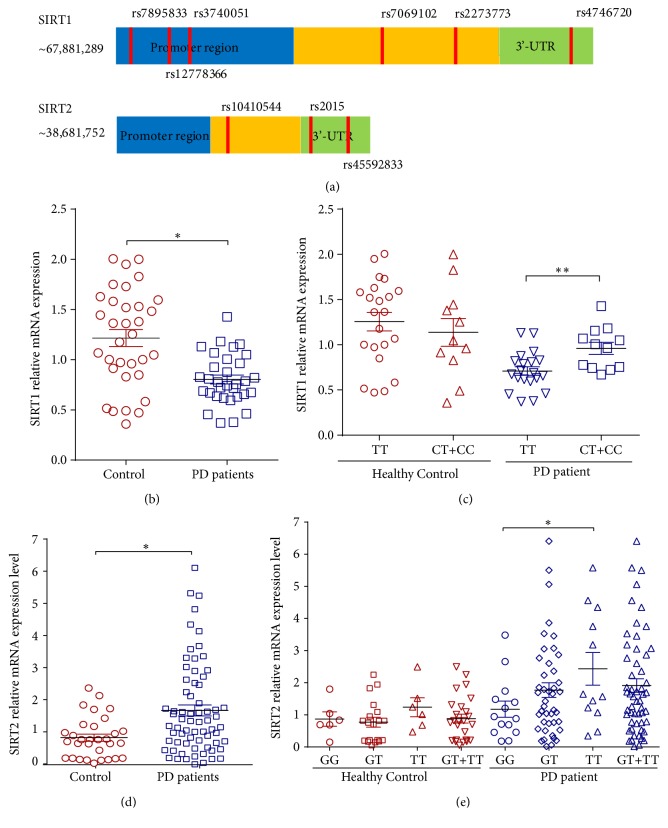
*The genotype distribution between the two SNPs and the expression of their corresponding sirtuins in the peripheral blood of PD patients and healthy controls.* (a) The distribution area of the nine SNPs in SIRT1 and SIRT2 gene. (b) The mRNA expression level of SIRT1 distribution in the peripheral blood of the PD patient (n=33) and healthy control (n=32). (c) The mRNA expression level of SIRT1 distribution of rs12778366 C/T genotype carriers in the peripheral blood of the PD patient and healthy control. (d) The mRNA expression level of SIRT2 distribution in the peripheral blood of the PD patient (n=31) and healthy control (n=68). (e) The mRNA expression level of SIRT2 and the distribution of rs2015 G/T genotype carriers in the peripheral blood of the PD patient and healthy control. Data represent the mean ± SEM, *∗*P<0.05 vs. control group, *∗∗*P<0.01 vs. control group.

In order to verify whether these two SNPs influence the corresponding SIRT1 or SIRT2 expression, we detected the expression level of SIRT1 and SIRT2 mRNA in peripheral blood samples extracted from the PD patients and healthy control. RNA extraction and quantitative real-time reverse transcription PCR were carried out regularly. As shown in Figure 1(b), we found that the SIRT1 mRNA expression levels in the peripheral blood of PD cases were significantly reduced relative to the controls. Opposite to the SIRT1, the SIRT2 mRNA expression levels in the peripheral blood of PD cases were apparently increased compared with the controls (Figure 1(d)). Moreover, on the basis of this data, we subdivided the cases and controls groups by genotype to dissect the relationship between SIRT1/2 level and the different genotype carriers. We observed that the C/T+C/C genotype carriers of rs12778366 showed an elevated expression level of SIRT1 compared with TT genotype carriers in PD patients (Figure 1(c)), and the T/T carriers of rs2015 showed an increased expression level of SIRT2 compared with GG carriers (Figure 1(e)). These results speculated that the SNPs rs12778366 and rs2015 may be functional by influencing the corresponding SIRT1 or SIRT2 expression.

**Figure 2:**
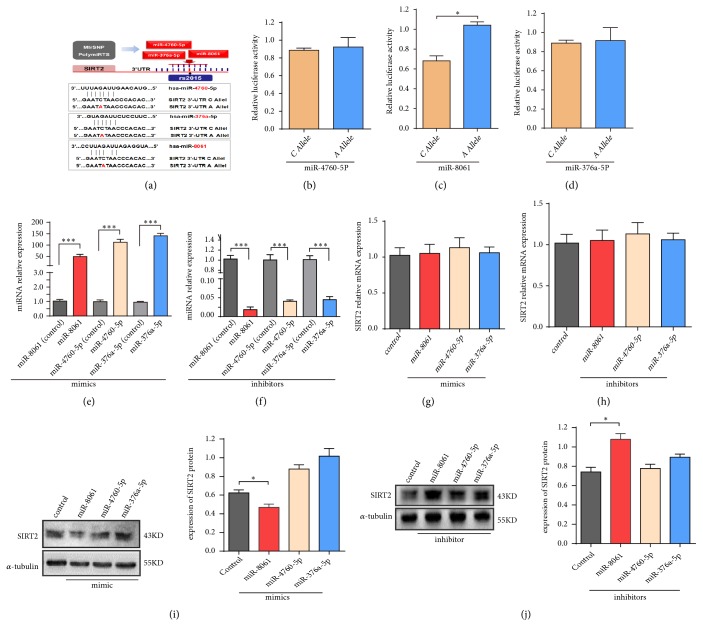
*The screening of the target miRNA on the 3*′*UTR within rs2015 of SIRT2 gene and the influence of rs2015 on the binding efficiency in vitro.* (a) Diagram of the prediction of the miRNA-binding site of SIRT2 3′UTR within SNP rs2015. (b, c, d) Luciferase reporter constructs containing SIRT2 3′UTR or its mutants were cotransfected with three corresponding miRNA mimics (miR-4760-5P, miR-8061, and miR-376a) in HEK-293T cells. (e, f) The expression level of corresponding miRNAs after transfected mimics or inhibitors of miRNAs to the SH-SY5Y cell by QRT-PCR assay, respectively. (g, h) The mRNA expression level of SIRT2 with the corresponding miRNAs' intervention in SH-SY5Y cell by QRT-PCR assay, respectively. (i, j) Western blot analysis for SIRT2 expression treated with mimics or inhibitors of the corresponding miRNA in SH-SY5Y cells. Data represent the mean ± SEM of three or four independent experiments. *∗*P<0.05 vs. control group, *∗∗*P<0.01 vs. control group, *∗∗∗*P<0.0001 vs. control group.

As rs2015 is located in the 3′UTR of the SIRT2 gene, we further investigated whether a possible miRNA binds the 3′UTR of SIRT2, influences the binding efficiency, then contributes to the change of SIRT2 expression levels. Three miRNAs, miR-376a-5p, miR-4760-5p, and miR-8061, targeting the binding site within the SNP rs2015 in SIRT2 3′UTR were predicted by the MirSNP and PolymiRTS database (Figure 2(a)). Then for evaluating which predicted miRNA located in the 3′UTR of SIRT2, the dual luciferase assay was performed in HEK-293T cells. As shown in Figure 2(c), when cotransfected with miR-8061 in HEK-293T cells, rs2015 C>A mutation increased the transcription activity of the luciferase reporter gene, indicating high binding ability of the C allele compared with A allele (Figure 2(c)). However, no significant change was observed in the transcription activity of the luciferase reporter gene when cotransfected with miR-4760-5p or miR-376a-5p (Figures 2(b) and 2(d)). Furthermore, we further evaluated whether these miRNAs influence the expression of SIRT2 mRNA in SH-SY5Y cells. Unexpectedly, the relative mRNA expression of SIRT2 did not differ after the mimics/inhibitors of the three miRNAs were transfected (Figures 2(e)–2(h)), suggesting that these miRNAs may not affect the SIRT2 expression in the transcription level. In addition, we assessed the expression level of SIRT2 protein under the interference of the three miRNAs, and miR-4760-5p and miR-376a-5p showed no significant association with SIRT2 protein expression whether the inhibitors or the mimics of the corresponding miRNAs were used, respectively. However, the miR-8061 inhibitors significantly upregulated the expression level of SIRT2 protein compared with the control (Figure 2(j)) and the mimics of miR-8061 also reduced the protein level of SIRT2 compared with the control (Figure 2(i)) in SH-SY5Y cells. Combining the results of miR-8061 in double luciferase assay, these results preliminarily indicate that rs2015 could influence the binding effect between miR-8061 and the 3′UTR region of SIRT2 gene, and miR-8061 could regulate SIRT2 protein expression level, which may ultimately have an effect on modulating PD pathology or risk.

## 4. Discussion

It is worth noting that the critical role that sirtuins played in PD progression is gradually being understood with a growing body of evidence shown in recent years [24]. As the important members of sirtuin family, SIRT1/2 have been reported to be closely associated with the process of PD, and more interestingly, SIRT1/2 have opposite effect on PD; either inhibition of SIRT2 or activation of SIRT1 has protective effect on PD [12]. However, despite the evidence in vitro and in vivo, less data are available on the role of SIRT1 and SIRT2 common genetic variants in PD risk. In the study, nine SNP in SIRT1/2 were selected to explore the association between these SNPs with PD susceptibility in Chinese Han population in 222 PD patients and 161 controls. Our results identified only rs12778366 in SIRT1 and rs2015 in SIRT2 are associated with PD in the Chinese Han population.

In this study, 9 representational SNPs located in the potential function regions of SIRT1/ SIRT2 or having been reported to be associated with several diseases were chosen. It has been reported that SIRT1 rs2273773 is associated with Rheumatoid Arthritis (RA), chronic obstructive pulmonary disease (COPD) [25]; rs7069102 is associated with COPD [25], cardiovascular diseases (CVD) [26], and myocardial infarction [27]; rs7895833 is associated with COPD [25], lifespan longevity [28], and obesity [29]; rs4746720 is associated with Type 2 Diabetes Mellitus [30]. All the above means SIRT1 SNPs have relationship with many diseases, but in our study, we do not observe these SNPs contribute to the risk of PD. A significant association was observed in the genotype and allele frequencies of the SIRT1 promoter SNP rs12778366 between the PD cases and controls. SIRT1 mRNA expression levels in the peripheral blood of PD cases were significantly reduced relative to the controls. Rs12778366 has been reported to increase IL-6 related human mortality [31], and the serum concentrations of IL-6 were higher in PD patients compared to the healthy controls [32–34]. Furthermore, the elevated level of IL-6 in peripheral blood may result from the SNP rs12778366 in SIRT1, suggesting that SNP rs12778366 may lead to SIRT1 protein overexpression and ultimately increase serum level of IL-6. However, it needs a direct measure of SIRT1 mRNA level in their samples, which may help assess this hypothesis and support a functional association between SIRT1 rs12778366 and SIRT1 transcription. On the other hand, compared to the subjects, the overall age of the sample in our study was younger, and different areas and living environment as well as age also have the effect on the regulation of gene phenotype. Anyway, the population-specific factors such as ethnic heterogeneity, population history, differences in genetic structure, environmental exposure, dietary, and cultures can influence the results that the same SNP site may show the different association with a disease; more in-depth evidence is still needed.

Considerable evidence has shown that SNPs located in the 3′UTR of genes have roles in the diagnosis, treatment outcome, and survival in human disease [35]. In context of PD, SNPs located in 3′UTR of FGF20, SNCA, PARKIN, LRRK2, CTSB, STX1B, IGSF9B, and HSD3B7 have been reported to be associated with Parkinson's disease through impact of their miRNA-binding strength and miRNA-mediated regulation of themselves [36–41]. Rs45592833, which was located in 3′UTR of SIRT2, has been identified to provide a significant association with human longevity in a case-control study of Calabrian origin [42]. But in our study, we did not observe the relationship between Rs45592833 and PD in our population. It has been investigated that rs2015, which is another SNP located in 3′UTR of SIRT2, is associated with susceptibility to colorectal cancer (CRC), and, the subjects with A allele were associated with lower CRC risk and higher SIRT2 expression in Chinese Han Beijing population compared with the C allele carriers[43]. This result was consistent with the research with the antitumor effects of SIRT2 on CRC [44]. Considering our research, comparing G carriers and T carriers of rs2015 showed increased expression of SIRT2 in the blood of PD patients, which according to the above-mentioned research means that rs2015 may be a certain functional SNP site influencing SIRT2 expression in Chinese Han population. Recently, SIRT2 inhibition has been firmly confirmed to reduce the formation of LBs and have a protective effect on PD pathology though increasing *α*-Syn acetylation and reducing *α*-Syn aggregation [23]. The finding also provides considerably potential therapeutic value of SIRT2 inhibition in PD treatment and in accordance with our result it showed that SIRT2 is overexpressed in PD.

In conclusion, our case-control study identified nine SNPs of SIRT1/ SIRT2 gene with PD in Chinese Han population for the first time and observed that two SNPs, rs12778366 and rs2015, were associated with the risk of PD in the Chinese Han population, and the rs2015 polymorphism may influence SIRT2 expression by affecting the binding of miR-8061 to the 3′UTR of SIRT2, ultimately contributing to the risk of PD. However, some limitation should be addressed in our study. Although we observed changes in the mRNA levels of sirtuins in the peripheral blood of PD and control patients, and the PD patients and healthy controls were recruited with the strict and unified inclusion criteria, the influence of SIRT1/SIRT2 levels by medication or other unrelated diseases even after standard exclusion of PD patients cannot be excluded in this study. Further studies should be focus on the further investigation of this association in larger sample size and exploration of the underling mechanism of the function of rs12778366 in PD risk.

## Figures and Tables

**Table 1:** The general clinical data of PD group and control group.

Clinical data	PD group (n=222)	Control group (n=161)	*P-*value
Age (year)	68.17±10.06	66.34 ±7.50	0.0581
male /female	134/88	89/72	0.3196

**Table 2:** Frequency distribution of nine SNPs of SIRT1 and SIRT2 gene in PD patients and healthy controls.

Gene	SNP	Genotype	Allele	Dominant model		Recessive model	
		P-value	P-value	P-value	95%CI	P-value	95%CI
SIRT1	rs3740051 G>A	0.0967	0.7481	0.6176	0.7381-1.667	0.0591	0.9504 - 5.346
SIRT1	rs2273773 C>T	0.1363	0.8568	0.0948	0.8729 - 4.670	0.1456	0.4919 - 1.111
SIRT1	rs7069102 G>C	0.4880	0.7627	0.5260	0.5312 - 1.382	0.5294	0.1484 - 2.291
SIRT1	rs12778366 C>T	0.0459	0.0111	0.1670	0.0999 - 1.328	**0.0221**	1.0760- 2.668
SIRT1	rs4746720 C>T	0.7823	0.7894	0.9012	0.6640 - 1.592	0.5460	0.7092 - 1.914
SIRT1	rs7895833 G>A	0.7928	0.7939	1.159	0.7724 - 1.741	0.5360	0.2646 - 1.086
SIRT2	rs2015 G>T	0.0431	0.4590	0.4155	0.7529 - 1.987	**0.0436**	1.0120 - 2.599
SIRT2	rs10410544 C>T	0.3097	0.3173	N.A	N.A	N.A	N.A
SIRT2	rs45592833 G>T	0.7928	0.7939	N.A	N.A	N.A	N.A

## Data Availability

The data used to support the findings of this study are available from the corresponding author upon request.

## Supplementary Materials

Supplementary Materials Supplement Table 1. The distribution of nine SNPs between PD patient and healthy control.
