# Supplementary material for: Rs2015 Polymorphism in miRNA Target Site of Sirtuin2 Gene Is Associated with the Risk of Parkinson's Disease in Chinese Han Population
Source: Biomed Res Int. 2019 May 12;2019:1498034. doi: 10.1155/2019/1498034 (PMC6535834; doi:10.1155/2019/1498034)
Supplement: Supplementary Materials — Supplement Table 1. The distribution of nine SNPs between PD patient and healthy control. [file 1498034.f1.doc]

Supplement table 1 . The distribution of nine SNPs between PD patient and healthy control.

| **rs12778366T>C** | **PD(n=222)** | **Control(n=161)** | **OR** | **95%CI** | **P-value** |
| --- | --- | --- | --- | --- | --- |
| **T/T** | **144** | **122** |  |  | **0.0459*** |
| **C/T** | **67** | **36** |  |  |  |
| **C/C** | **11** | **3** |  |  |  |
| **C/T+C/C** | **78** | **39** | **1.694** | **1.076 to 2.668** | **0.0221*** |
| **T/T+C/T** | **211** | **158** | **0.3642** | **0.09991 to 1.328** | **0.1670** |
| **T** | **355** | **280** | **0.5983** | **0.4014 to 0.8917** | **0.0111*** |
| **C** | **89** | **42** |  |  |  |
| **rs7895833G>A** | **PD(n=222)** | **Control(n=161)** | **OR** | **95%CI** | **P-value** |
| **G/G** | **109** | **85** |  |  | **0.214** |
| **G/A** | **84** | **64** |  |  |  |
| **A/A** | **29** | **12** |  |  |  |
| **G/A+A/A** | **113** | **76** | **1.159** | **0.7724 to 1.741** | **0.4751** |
| **G/A+G/G** | **193** | **149** | **0.536** | **0.2646 to 1.086** | **0.0796** |
| **G** | **302** | **234** | **0.7998** | **0.5831 to 1.097** | **0.1655** |
| **A** | **142** | **88** |  |  |  |
| **rs3740051A>G** | **PD(n=222)** | **Control(n=161)** | **OR** | **95%CI** | **P-value** |
| **A/A** | **117** | **89** |  |  | | **0.0967** | | --- | |
| **G/G** | **9** | **14** |  |  |  |
| **G/A** | **96** | **58** |  |  |  |
| **G/A+A/A** | **213** | **147** | **2.254** | **0.9504 to 5.346** | **0.0591** |
| **G/A+G/G** | **105** | **72** | **1.109** | **0.7381 to 1.667** | **0.6176** |
| **G** | **114** | **86** | **0.948** | **0.6842 to 1.313** | **0.7481** |
| **A** | **330** | **236** |  |  |  |
| **rs2273773T>C** | **PD(n=222)** | **Control(n=161)** | **OR** | **95%CI** | **P-value** |
| **T/T** | **116** | **89** |  |  | **0.1363** |
| **C/T** | **96** | **58** |  |  |  |
| **C/C** | **10** | **14** |  |  |  |
| **C/T+C/C** | **106** | **72** | **0.7392** | **0.4919 to 1.111** | **0.1456** |
| **T/T+C/T** | **212** | **147** | **2.019** | **0.8729 to 4.670** | **0.0948** |
| **T** | **328** | **236** | **1.03** | **0.7443 to 1.427** | **0.8568** |
| **C** | **116** | **86** |  |  |  |
| **rs7069102 G＞C** | **PD(n=222)** | **Control(n=161)** | **OR** | **95%CI** | **P-value** |
| **C/C** | **173** | **121** |  |  | **0.4880** |
| **G/C** | **42** | **37** |  |  |  |
| **G/G** | **7** | **3** |  |  |  |
| **G/C+G/G** | **49** | **40** | **0.8568** | **0.5312 to 1.382** | **0.5260** |
| **G/C+C/C** | **215** | **158** | **0.5832** | **0.1484 to 2.291** | **0.5294** |
| **G** | **56** | **43** | **0.9365** | **0.6115 to 1.434** | **0.7627** |
| **C** | **388** | **279** |  |  |  |
| **rs4746720 C＞T** | **PD(n=222)** | **Control(n=161)** | **OR** | **95%CI** | **P-value** |
| **C/C** | **44** | **36** |  |  | **0.7823** |
| **C/T** | **109** | **74** |  |  |  |
| **T/T** | **69** | **51** |  |  |  |
| **C/T+C/C** | **153** | **110** | **1.028** | **0.6640 to 1.592** | **0.9012** |
| **T/T+C/T** | **178** | **125** | **1.165** | **0.7092 to 1.914** | **0.5460** |
| **C** | **197** | **146** | **0.9615** | **0.7205 to 1.283** | **0.7894** |
| **T** | **247** | **176** |  |  |  |
| **rs45592833 G＞T** | **PD(n=222)** | **Control(n=161)** | **OR** | **95%CI** | **P-value** |
| **GG** | **217** | **158** |  |  | **0.7928** |
| **GT** | **5** | **3** |  |  |  |
| **TT** | **0** | **0** |  |  |  |
| **G** | **439** | **319** | **0.8257** | **0.1958 to 3.481** | **0.7939** |
| **T** | **5** | **3** |  |  |  |
| **rs2015 G＞T** | **PD(n=222)** | **Control(n=161)** | **OR** | **95%CI** | **P-value** |
| **GG** | **45** | **47** |  |  | **0.0431*** |
| **GT** | **131** | **75** |  |  |  |
| **TT** | **46** | **39** |  |  |  |
| **GG+GT** | **176** | **122** | **1.223** | **0.7529 to 1.987** | **0.4155** |
| **GT+TT** | **177** | **114** | **1.622** | **1.012 to 2.599** | **0.0436*** |
| **G** | **221** | **169** | **0.8972** | **0.6732 to 1.196** | **0.4590** |
| **T** | **223** | **153** |  |  |  |
| **rs10410544 C＞T** | **PD(n=222)** | **Control(n=161)** | **OR** | **95%CI** | **P-value** |
| **T/T** | **0** | **0** |  |  | **0.3097** |
| **C/C** | **211** | **149** |  |  |  |
| **C/T** | **11** | **12** |  |  |  |
| **C** | **433** | **310** | **1.524** | **0.6636 to 3.499** | **0.3173** |
| **T** | **11** | **12** |  |  |  |
